# Achilles Tendon Softness and Thickness in Patients With Hypercholesterolemia

**DOI:** 10.7759/cureus.28340

**Published:** 2022-08-24

**Authors:** Arul Murugan, Kirubhakaran Kanakaraju, Sidesh R M, Vandana Sanjoy Mishra

**Affiliations:** 1 General Medicine, Vinayaka Mission's Kirupananda Variyar (VMKV) Medical College, Salem, IND

**Keywords:** hyperlipidemia, cardiovascular disease, achilles tendon thickness and softness, hypercholesterolemia, achilles tendon xanthomas

## Abstract

Background

Hypercholesterolemia is a condition where blood levels of cholesterol are high. It is of two types: The first type is familial hypercholesterolemia, which is hereditary, and the second one is due to diseases like diabetes, thyroid, etc. Achilles tendon xanthomas are noted in both types of hypercholesterolemia, which can be used as an indicator that predicts early cardiovascular disease. The aim of the study is to estimate the Achilles tendon thickness (ATT) and softness among hypercholesterolemia patients and to find the correlation between ATT and total cholesterol.

Methodology

A hospital-based cross-sectional, analytical study was done in a tertiary care hospital, Salem, for eight months. Patients of age over 18 years of both sexes who came for screening of total cholesterol in the outpatient department were included in the study. Those patients with a history of previous leg injury involving the Achilles tendon were excluded from the study. A pre-structured questionnaire was used to collect the data, and analysis was done using Statistical Package for the Social Sciences (SPSS) v20 (IBM Corp., Armonk, NY). The analysts performed the Pearson correlation test to determine the correlation between two continuous variables. A p-value of less than 0.05 was used to indicate statistical significance.

Results

In this study, there are 40 participants in the normal group and about 60 participants in the secondary hypercholesterolemia group. The mean ATT value among males and females was 9.3 and 6.1 mm, respectively. A positive correlation was noted between the ATT and total cholesterol value (p-value = 0.0001).

Conclusion

The thickness and softness of the Achilles tendon are positively correlated with the serum total cholesterol level. Males are the group where this correlation is most significant. As a result, men have a higher risk of developing Achilles tendon thickening than women. The thickness of the Achilles tendon can therefore be one of the early signs of high cholesterol levels. The clinician can utilize this indicator to evaluate early abnormal cardiac illness.

## Introduction

The Achilles tendon is the strongest and thickest tendon in our body [1]. In disorders of lipid metabolism and familial hypercholesterolemia (FH), xanthomas appear in the Achilles tendon. Tendinous xanthoma can be a clinical sign that manifests early in the disease's progression [2,3]. Xanthomas are comprised of monocyte-derived foam cells and lipids [4]. Lipids may deposit at later stages of the disease in hypercholesterolemia, which is because of diseases like type-2 diabetes and hypothyroidism. Patients with premature acute coronary syndrome (ACS) who were admitted to Japan are found to have FH, which highlights the significance of early detection and treatment of this condition [5].

The earliest sign of Achilles tendon xanthoma is the thickening of the tendon, which is seen through digital radiography [6,7]. Achilles tendon thickening can be a risk factor for cardiovascular disease [8]. Recently, ultrasonography was widely used to evaluate the Achilles tendon thickness (ATT) in Western countries [9]. As ultrasound is a simple and non-invasive procedure, we measure not only its thickness but also internal properties such as width and area. Sufficient studies describe the role of ultrasound-based ATT [10-12].

Thickening of the tendon, loss of echo texture, and gaps can be recognized by comparing with healthy individuals [13]. In elastography, a healthy tendon will appear firm, while the ruptured one and the one with tendinopathy will be softer [14-16]. With this background, our goal was to measure the softness and thickness of the Achilles tendon in patients and assess its association with hypercholesterolemia.

## Materials and methods

Study setting

The current study is a cross-sectional, analytical study. This study was recorded for eight months, from August 2021 to March 2022, in the Internal Medicine Department of a tertiary care hospital in Salem, Tamil Nadu.

Sample size

This study included all patients who visited the Internal Medicine Department’s outpatient clinic at a tertiary care facility in Salem during the study period (universal sampling). The participants in the study were individuals who provided their informed consent. In our study, about 100 individuals participated. The minimal sample size required for the study with a 95% confidence interval was 100, assuming the prevalence of hypercholesterolemia was 50% with an absolute error of 10%. The sample size was calculated using the formula 3.86*p*q/d^2^, where p stands for prevalence, q stands for a complement of p, and d stands for absolute precision.

Inclusion criteria

The study included all patients over the age of 18 years who went to the outpatient department for a total cholesterol screening.

Exclusion criteria

The excluded conditions are tenosynovitis, tendinitis, bursitis, tuberculum arthritis, and rheumatoid arthritis since they could affect the ATT. Patients who underwent surgery on their Achilles tendons, sustained injuries to their tendons, or took statin-type drugs to decrease their cholesterol were excluded from the study.

Ethical clearance

The Institutional Ethics Committee of Vinayaka Mission’s Kirupananda Variyar Medical College and Hospital (VMKVMC&H), Salem, gave its approval before beginning the current study (VMKVMC&H/IEC/21/101).

Data collection

The collected data comprise baseline characteristics like name, age, sex, and comorbid status from the structured questionnaire. The patient information sheet offered values on total cholesterol level, low-density lipoprotein (LDL), and triglyceride values.

The researchers also considered blood parameters like fasting blood sugar, postprandial blood sugar, total cholesterol, low-density lipids, and triglycerides. All the patients underwent B-mode ultrasonography to measure the thickness of the Achilles tendon. Investigators instructed the patients to lie face down with their ankles dorsiflexed. Then, a 10 MHz or a higher frequency probe when used longitudinally measured the thickness. The normal thickness should be less than 6 mm. Any measurement more than that indicates thickening of the tendon.

The investigators measured the elastographic images qualitatively by visual inspection through the color map pattern. The hard tendon fibers appeared as blue, intermediate softening as yellow and green, and marked softening by red color.

According to the criteria given in Klauser et al.'s study [14], we grade it as follows - Grade 1: blue (hardest) to green (hard); Grade 2: yellow (soft); and Grade 3: red (softest). Grades 2 and 3 are pathological tendons.

Statistical analysis

The analysts entered the collected data in MS excel of Windows 10. We used Statistical Package for the Social Sciences (SPSS) version 20 (IBM Corp., Armonk, NY) to conduct our statistical analysis. We expressed all the continuous variables in terms of mean and standard deviation and all the categorical variables in terms of numbers and percentages. The analysts used the Pearson correlation method was used to determine the correlation between two continuous variables. We defined the statistical significance as a p-value less than 0.05.

## Results

Among the study participants, 40% presented with a normal level of cholesterol, and the remaining 60% presented with elevated or abnormal cholesterol. The mean age of the normal cholesterol group was 53.14 years and that of the abnormal or elevated cholesterol group was 55.14 years. There were 22 patients with diabetes and 12 with hypertension in the normal cholesterol group, whereas 34 with diabetes and 20 with hypertension in the abnormal or elevated cholesterol group. The mean LDL value is higher in the abnormal or elevated cholesterol group (147 mg/dl) when compared to the normal cholesterol group (140 mg/dl). The mean total cholesterol and triglycerides were high in the abnormal cholesterol group when compared to the normal group. Table 1 describes the clinical and baseline characteristics of the study participants.

**Table 1:** Baseline characteristics of the study population

Variables	Normal cholesterol level (N = 40)	Abnormal cholesterol level (N = 60)
Age (years)	53.14 ± 9.6	55.14 ± 10.82
Sex (M:F)	27/23	34/30
Height (cm)	160.61 ± 6.4	161.72 ± 8.26
Weight (kg)	58.00 ± 10.28	61.55 ± 10.25
BMI (kg/m^2^)	22 ± 3.00	25 ± 4.00
Systolic blood pressure (mmHg)	127.16 ± 11.95	133.62 ± 15.78
Diastolic blood pressure (mmHg)	80.89 ± 10.32	84.22 ± 10.02
Total cholesterol (mg/dl)	215 ± 60	224 ± 56
Low-density lipoprotein-C (mg/dl)	140 ± 48	147 ± 54
High-density lipoprotein-C (mg/dl)	52 ± 16	54 ± 17
Triglycerides (mg/dl)	98 (60-118)	120 (83-174)
Comorbidities		
Diabetes	22	34
Hypertension	12	20
Asthma	6	6

When compared between males and females, the males have high ATT, area, and width among the abnormal cholesterol group with statistical significance using an independent T-test. Table 2 depicts the relationship between these two.

**Table 2:** Sex differences in the Achilles tendon among the abnormal cholesterol level of study participants

Achilles tendon	Male	Female	P-value
Thickness (mm)	9.3 (5.6-11.60)	6.1 (5.7-9.6)	<0.001
Width (mm)	17.5 (14-21)	13.2 (12.8-17.8)	<0.001
Area (mm)	1.60 (0.63-1.84)	0.27 (0.52-1.44)	<0.001

Considering the elastography results, Fisher’s exact test shows a significant statistical association between the abnormal cholesterol level and the middle and distal tendon segments grading two and three with a p-value of less than 0.001. No significant difference was found in the proximal portion of the tendon between the normal and abnormal cholesterol groups. Table 3 depicts the relationship between these abnormal cholesterol levels and the elastography findings.

**Table 3:** Ultra-elastography grading of the Achilles tendon segments in patients with abnormal cholesterol levels

Elastography findings	Normal cholesterol level (40)	Abnormal cholesterol level (60)	P-value
Proximal			
Grade 1	40	57	0.075
Grade 2	0	3	
Grade 3	0	0	
Sum of grade abnormality (2+3)	0	3	
Middle			
Grade 1	35	36	<0.001
Grade 2	2	14	
Grade 3	3	10	
Sum of grade abnormality (2+3)	5	24	
Distal			
Grade 1	38	43	<0.001
Grade 2	2	11	
Grade 3	0	6	
Sum of grade abnormality (2+3)	2	17	

As the serum total cholesterol level increases, there is a marked thickening in the Achilles tendon among the study participants with a positive correlation. Hence, we considered this association statistically significant. Table 4 illustrates the correlation between the ATT and the total cholesterol level.

**Table 4:** Correlation between the Achilles tendon thickness and total cholesterol

Pearson’s correlation		Total cholesterol
Achilles tendon thickness	Correlation coefficient	0.701
	P-value	<0.001
	N	100

As the serum total cholesterol level increases, there is a marked softening in the Achilles tendon among the study participants with a good positive correlation. Hence, we considered this association statistically significant. Table 5 illustrates the correlation between the Achilles tendon softness and the total cholesterol level.

**Table 5:** Correlation between the Achilles tendon softness and total cholesterol

Spearman’s correlation		Total cholesterol
Achilles tendon softness	Correlation coefficient	0.723
	P-value	<0.001
	N	100

## Discussion

This study included 60 people having abnormal or increased cholesterol levels and 40 participants having normal cholesterol levels. The current study found that the mean age of the normal cholesterol group was 53.14 years and that of the abnormal or elevated cholesterol group was 55.14 years. We observed that the Achilles tendon is a common site for lipid deposition. This characteristic may serve as a preliminary sign of xanthoma development.

Our study aims to compare the ATT with the baseline and clinical features of the normal cholesterol level group and the abnormal or elevated cholesterol level group. We noticed a similar observation in a study conducted by Wang et al. in China [16]. In this study, they could not find an association of the ATT among males or females with high cholesterol levels.

In our study, we measured a 9.3-mm ATT in men and 6.1-mm ATT in women. The independent T-test reveals a statistically significant difference between them. Thus, our study shows that there is a correlation between the ATT and high cholesterol levels in males. These observations are comparable to the results in the study by Michikura et al. (2017) in Japan [17].

We found elastography to be an effective method in finding the softness of the Achilles tendon compared to x-ray and MRI. Our study found that the Achilles tendon is harder in the abnormal cholesterol group when compared to the normal cholesterol level group with a statistical significance.

In our study, the group with abnormal cholesterol had a higher proportion of participants in Grades 2 and 3. These findings are comparable to the observations of the study by El Badry et al. [18]. They found that the hypercholesterolemia patients have a greater number of Grade 2 and Grade 3 findings with soft Achilles tendon, which is in contrast to our study's findings. In contrast to the above finding, a study conducted by Sconfienze et al., [19] found that symptomatic patients have harder tendons compared to normal persons. This difference in the results among various studies could be because of inconsistent techniques or different grading systems used.

Similarly, in our study, the middle portion of the tendon is most involved, followed by the distal and proximal portions. These findings are comparable to the studies by van Dijik et al. [20] and Maffulli et al. [21], in which similar results are observed.

We found a positive correlation with a statistical significance between the total cholesterol and Achilles tendon thickness and also between total cholesterol and Achilles tendon softness. We also found these correlations with the abnormal or elevated cholesterol group.

Limitation of the study

Despite the fact that the current study found a correlation between thick Achilles tendons and high serum cholesterol, the study's limited sample size prevented the estimation of the ATT's predictive value for serum cholesterol value. Since the study was conducted in a hospital, it cannot be generalized to the general population. The external validity of the study would be guaranteed by a multicentric or community-based study.

## Conclusions

Both ATT and softness have a positive correlation with the serum total cholesterol level. This correlation is most significant among males. This indicates that male gender is a high-risk population showing more ATT than females. Thus, one of the early indicators of elevated cholesterol levels can be the ATT. To evaluate early abnormal cardiac problems, the clinician can use this indicator. This study laid a foundation for more diagnostic studies to set a cut-off value. The physician can use ATT as a cost-effective screening or diagnosing tool.
